# Clinical Notes

**Published:** 1893-06

**Authors:** William Steinrauf

**Affiliations:** St. Charles, Mo.


### CLINICAL NOTES.

William Steinrauf, M. D., St. Charles, Mo.

Was called, some months ago, to see Mrs. Tynen, who was about to be confined with her first child. Found her husband and two old ladies in attendance, urging her on. The pains occurred every few minutes, but seemed to be of short duration. After watching her a little while I thought I saw an indication for Gelsemium. She took it, but it did no good. Belladonna was given with the same result, and also Cimicifuga. Concluded to give nothing, and let matters take their own course.

As I could not find the indicated remedy from the condition of the uterus I concluded to watch the mental condition. I must not forget to add that the lady was a perfect stranger to me. And such irritability was enough to distract anybody. Her snappish, cross actions were beyond endurance. She was uncivil and spiteful in the extreme. Chamomilla$^{dmm}$, one dose, and the pains changed within five minutes. She acted like a different woman. Began to treat her husband and all present with more kindness and gentleness, and in about one-half hour the labor was over. The case taught me a valuable lesson.

Was asked to see Mrs. B. a few nights ago, who had been confined a day before by a midwife. The labor had been a normal one. She complained of fever, headache, and general restlessness. Aconite$^{3x}$ in water and one teaspoonful every half-hour till better, and then less frequently. No better. With Belladonna I had the same result. Now a severe diarrhœa set in, and I became somewhat alarmed.

The remedies had proven valueless so far. I did not, on that account, blame Homœopathy, but simply and naturally blamed myself.

Set to work now and studied the case thoroughly, as I should have done in the start.

Pulse 110, weak and feeble; skin only moderately hot; diarrhœa every half-hour; headache with giddiness and faintness. She feels so utterly without power. Thinks she would be well if the neighbors had not tried to frighten and scare her about family affairs.

Gelsemium$^{3x}$ in water cured intwelve hours. When this mental condition met its medicinal equal the diseased condition was wiped out.

